# Supplementary material for: Hybrid Models and Biological Model Reduction with PyDSTool
Source: PLoS Comput Biol. 2012 Aug 9;8(8):e1002628. doi: 10.1371/journal.pcbi.1002628 (PMC3415397; doi:10.1371/journal.pcbi.1002628)
Supplement: Text S4 — Complete source code for the PyDSTool package (version 0.88.120504). Includes API documentation and help files linking to web pages. This file is identical to the current public release on Sourceforge.net. (ZIP) [file pcbi.1002628.s004.zip › PyDSTool/html/PyDSTool.Toolbox.optimizers.step.quasi_newton_step-pysrc.html]

xml version="1.0" encoding="ascii"?


PyDSTool.Toolbox.optimizers.step.quasi\_newton\_step


| Home | Trees | Indices | Help | PyDSTool |
| --- | --- | --- | --- | --- |

Package PyDSTool ::
Package Toolbox ::
Package optimizers ::
Package step ::
Module quasi\_newton\_step

[hide private]

[frames] | no frames]

# Source Code for Module PyDSTool.Toolbox.optimizers.step.quasi\_newton\_step

```
 1   
 2  # Matthieu Brucher 
 3  # Last Change : 2007-08-30 09:23 
 4   
 5  """ 
 6  Computes a quasi-Newton step for a specific function at a specific point 
 7  """ 
 8   
 9  import numpy 
10  import numpy.linalg 
11   


12 -class DFPNewtonStep(object):


13    """ 
14    The Davidson-Fletcher-Powell Quasi-Newton step 
15    """ 


16 -  def __init__(self, hessian_approx):


17      """ 
18      Construct a DFP module 
19        - hessian_approx is an approximation of the hessian around the starting point 
20      """ 
21      self.B0 = numpy.linalg.inv(hessian_approx)

22   


23 -  def __call__(self, function, point, state):


24      """ 
25      Computes a gradient step based on a function and a point 
26      """ 
27      if 'Bk' not in state: 
28        Bk = self.B0.copy() 
29        gradient = function.gradient(point) 
30      else: 
31        Bk = state['Bk'] 
32        oldParams = state['old_parameters'] 
33        newParams = state['new_parameters'] 
34        gradient = function.gradient(point) 
35        oldGradient = state['gradient'] 
36   
37        yk = gradient - oldGradient 
38        sk = newParams - oldParams 
39        rho = 1/numpy.dot(yk.T, sk) 
40        Bk = numpy.dot(numpy.eye(len(gradient), len(gradient)) - rho * numpy.dot(yk[:, numpy.newaxis], sk[numpy.newaxis,:]),  numpy.dot(Bk, numpy.eye(len(gradient), len(gradient)) - rho * numpy.dot(sk[:, numpy.newaxis], yk[numpy.newaxis,:]))) + rho * numpy.dot(yk[:, numpy.newaxis], yk[numpy.newaxis,:]) 
41   
42      step = -numpy.dot(Bk, gradient) 
43   
44      state['Bk'] = Bk 
45      state['gradient'] = gradient 
46      state['direction'] = step 
47      return step

48
```

  


| Home | Trees | Indices | Help | PyDSTool |
| --- | --- | --- | --- | --- |

|  |  |
| --- | --- |
| Generated by Epydoc 3.0.1 on Fri May 4 15:24:24 2012 | http://epydoc.sourceforge.net |
